# 

**Published:** 1985-04

**Authors:** 


					Contents
Editorial
31.
Erythema Infectiosum?A Bristol Out-
break
C. R. Lovell
33.
Itch in Psoriasis
Clive E. Grattan
36.
Mrs Gillick and the Wisbech Area
Health Authority
John Griffiths
37.
Chronic Granulomatous Cicatrising
Enteritis?A clinical comparison of
Crohn's disease and Tuberculosis
M. M. Guirgis
39.
From our Correspondents
43.
From our Foreign Correspondents
Bangladesh?1977 to 1984
Arthur L. tyre-Brook
46.
Medical Officer in the Transkei
Andrew Farkas
51
Liver and Pancreas Transplantation
Report on the Marjorie Budd Lecture
by Professor R. Y. Calne
54.
Wessex Branch of the Association of
Clinical Pathologists
Abstracts of Meeting in Bristol,
November 1984
55.
South West Orthopaedic Club
Abstracts of Meeting in Exeter,
November 1984
58.
60. University of Bristol
Examination Results

				

